# Using lysis therapy to treat five critically ill COVID‐19 patients who show echocardiographic criteria of right ventricular strain

**DOI:** 10.1002/jha2.307

**Published:** 2021-10-13

**Authors:** Ahmed Mahdy, Eslam Abbas, Rawad Tarek, Nawar Jabbour, Huda Al‐Foudri

**Affiliations:** ^1^ Department of Cardiology Al Adan Hospital Kuwait City Kuwait; ^2^ Department of OncoCardiology Dar El Salam Cancer Center Cairo Egypt; ^3^ Department of Intensive Care Al Adan Hospital Kuwait City Kuwait

**Keywords:** COVID‐19, hemodynamic instability, lysis therapy, mechanical ventilation

## Abstract

New options for treatment escalation in critically ill COVID‐19 patients are unmet need. Five critically ill patients were treated using a low‐dose protocol thrombolytic therapy in the form of intravenous alteplase infusion over 15 min (0.6 mg/kg). This therapeutic intervention yielded an immediate favorable outcome on follow up and all five patients were extubated and transferred to the ward. This report suggests that bedside echocardiography can be a beneficial tool in the urgent assessment of the right ventricle—to—pulmonary vascular coupling in mechanically ventilated COVID‐19 patients, and thrombolytic therapy may be beneficial in hemodynamically unstable patients who show echocardiographic criteria of right ventricular strain.

## 1 INTRODUCTION

The case‐fatality rate of COVID‐19 patients characterized clinically as mild is 0%, while it reaches 50% in those patients who are clinically characterized as critical [1, 2]. These numbers indicate a critical point in the pathogenesis of COVID‐19 against which the current therapeutic approach proves to be ineffective. Over the past several months, our understanding of the disease has been changed by multiple landmark achievements that highlight the role of COVID‐induced hypercoagulability in patients’ outcomes [3, 4, 5, 6]. Coagulopathy associated with COVID‐19 may be driven by the production of prothrombotic autoantibodies [7] in addition to microthrombogenic responses that occur when endothelial insult takes place [8]. This reaction is aggravated causing enhanced platelet activation [9], and the microthrombotic pathway is maintained by the activation of both the coagulation factors and the endothelium [6]. Therefore, in situ immune‐thrombosis plays a significant role as a primary mechanism explaining the micro‐ and macrothrombotic manifestations of the disease [10]. Additionally, the COVID‐19 patients have additional risk factors for increased thrombosis such as hypoxia, and immobility in prone positioning [11, 12, 13].

The purpose of this study was to evaluate the clinical effects of using lysis therapy, to unplug the pulmonary vasculature, administered to mechanically ventilated, hemodynamically unstable COVID‐19 patients depending on the noninvasive echocardiographic assessment of the right ventricle‐to‐pulmonary vascular coupling.

## 2 METHODS IN BRIEF

Laboratory‐confirmed COVID‐19 patients who had any of the following were considered in a critical condition: (i) shock, identified by implementation of vasopressor therapy and elevated lactate levels (> 2 mmol/L) despite adequate fluid resuscitation, (ii) respiratory failure requiring mechanical ventilation, or (iii) failure of other organs necessitating admission to the intensive care unit.

### 2.1 Patients

COVID‐19 patients were eligible to receive lysis therapy if they fulfilled the following criteria: (i) had a rapidly progressive severe pneumonia, (ii) were currently supported by mechanical ventilation, (iii) PaO_2_/FiO_2_ ratio < 300 (wherein PaO_2_ measured in mmHg and FiO_2_ is the fraction of inspired oxygen expressed as a decimal), and (iv) the echocardiographic examination showed one or more criterion of right ventricular strain.

### 2.2 Lysis therapy

A low‐dose protocol of recombinant tissue‐type plasminogen activator [14] was implemented, wherein Alteplase (Activase®, Genentech) was intravenously infused over 15 min with a dose of 0.6 mg/kg (the maximum dose is 50 mg) [15].

## 3 RESULTS

Five patients (age range, 40–83 years; 1 woman) were treated with lysis therapy. None of whom was a smoker, and three patients had preexisting medical conditions in the form of diabetes mellitus, hypertension, and ischemic cardiomyopathy. All five patients had received tocilizumab (within 3 days of hospitalization) and low‐dose steroids without antiviral treatments (Table 1).

**TABLE 1:** Characteristics of COVID‐19 patients who received lysis therapy

	Patients				
	1	2	3	4	5
Age	49	40	53	83	70
Sex	M	M	M	F	M
Weight	81	64	87	78	73
Smoking	No	No	No	No	No
Comorbid diseases	None	None	DM	DM	IHD
			HTN	HTN	HTN
Disease presentation	ARDS	ARDS	ARDS	ARDS	ARDS
	MODS	MODS	MODS	MOF	MODS
Clinical classification	Critical	Critical	Critical	Critical	Critical
Treatment					
Antivirals	None	None	None	None	None
Antibiotics	Yes	Yes	Yes	Yes	Yes
Steroids	Dexamethasone	Methylprednisolone	Dexamethasone	Dexamethasone	Dexamethasone
Tocilizumab*	Yes	Yes	Yes	Yes	Yes
Anticoagulant**	Yes	Yes	Yes	Yes	Yes
Status***	Extubated, transferred to ward	Extubated, transferred to ward	Extubated, transferred to ward	Extubated, transferred to ward	Extubated, transferred to ward
Total length of hospital stay, d	40	62	25	50	58

M, male; F, female, DM, diabetes mellites; HTN, hypertension; ARDS, acute respiratory distress syndrome; MODS, multiorgan dysfunction syndrome; MOF, multiorgan failure.

* Tocilizumab was received as a single IV dose of 8 mg/kg (max 800 mg) within 3 days of hospitalization.

** Anticoagulation therapy was administered in a prophylactic dose.

*** All patients continued to receive oxygen therapy at the ward, and Patient 5 was discharged from the hospital on home oxygen.

All five patients were mechanically ventilated at the time of lysis therapy administration, all of them were weaned from mechanical ventilation and extubated postlysis (Table 1). Patient 5 was receiving ECMO at the time of lysis therapy administration but did not require ECMO within 6 days postlysis. All patients were transferred to the ward and eventually discharged from the hospital; length of stay was 40, 62, 25, 50, and 58 days, respectively (Patient 5 discharged on home oxygen due to pulmonary fibrosis).

### 3.1 Change in hemodynamics

**FIGURE 1:**
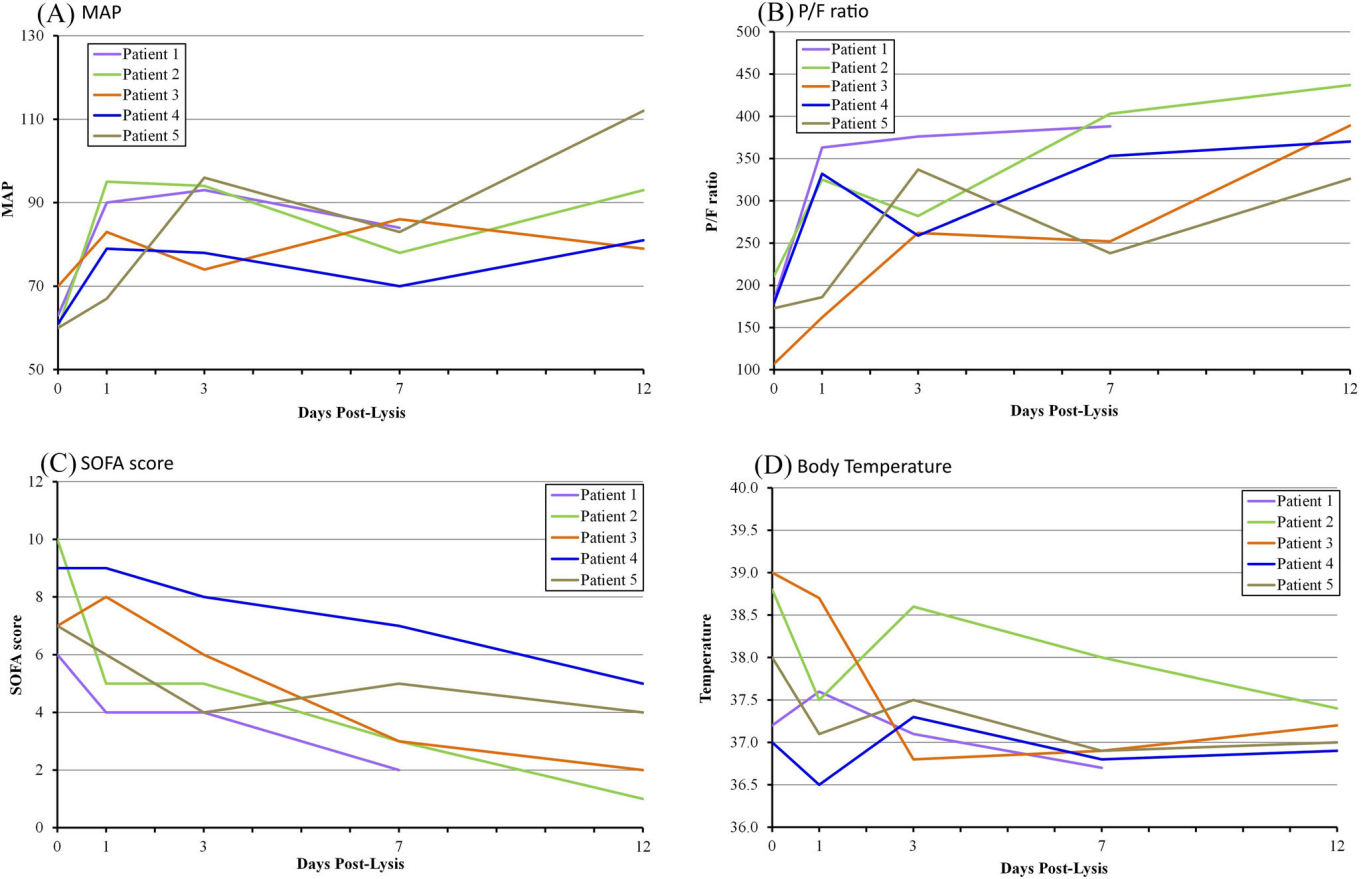
Temporal changes of mean arterial pressure (MAP), PAO2/FIO2 ratio, SOFA score, and body temperature in patients receiving lysis therapy. Wherein (A) change in mean arterial blood pressure (lowest value during the day was recorded) from day 0 to day 12 postlysis. (B) Change in PAO2/FIO2 ratio of the treated patients from day 0 to day 12 after treatment. (C) Represent change in Sequential Organ Failure Assessment (SOFA) score of the patients during the same duration (range 0–24, with higher scores indicating more severe illness). (D) Change in body temperature of the 5 patients prior to and postlysis

At the time of lysis therapy administration, all patients were hemodynamically unstable on circulatory support in the form of norepinephrine plus or minus vasopressin. The resultant mean arterial blood pressure (MAP) ranged from 60 to 70 mmHg. Upon administration of lysis therapy, all patients were gradually weaned from the circulatory support, and the MAP gradually increased over 80 mmHg within a time period that ranged from 7 to 12 days (Table 2, Figure 1A).

**TABLE 2:** Clinical indices, circulatory support, and laboratory results of COVID‐19 patients before and after lysis therapy

	Patients				
	1	2	3	4	5
Clinical indices					
MAP					
Day 0 before lysis	63	60	70	61	60
Day 1 postlysis	90	95	83	79	67
Day 3 postlysis	93	94	74	78	96
Day 7 postlysis	84	78	86	70	83
Day 12 postlysis		93	79	81	112
P/F ratio					
Day 0 before lysis	182	211	107	179	173
Day 1 postlysis	363	325	162	332	186
Day 3 postlysis	376	282	262	259	337
Day 7 postlysis	388	403	252	353	238
Day 12 postlysis		437	389	370	326
SOFA score					
Day 0 before lysis	+6	+10	+7	+9	+7
Day 1 postlysis	+4	+5	+8	+9	+6
Day 3 postlysis	+4	+5	+6	+8	+4
Day 7 postlysis	+2	+3	+3	+7	+5
Day 12 postlysis		+1	+2	+5	+4
Body temperature (°C)					
Day 0 before lysis	37.2	38.8	39	37	38
Day 1 postlysis	37.6	37.5	38.7	36.5	37.1
Day 3 postlysis	37.1	38.6	36.8	37.3	37.5
Day 7 postlysis	36.7	38	36.9	36.8	36.9
Day 12 postlysis		37.4	37.2	36.9	37
Circulatory support					
Norepinephrine (mcg/kg/h)					
Day 0 before lysis	0.3	0.18	0.8	0.2	0.5
Day 1 postlysis	0.2	0.1	0.3	0.4	0.15
Day 3 postlysis	0.08	0.02	0.3	0.05	0.05
Day 7 postlysis	0	0.02	0.01	0	0.04
Day 12 postlysis		0	0	0	0
Dobutamine (mcg/kg/min)					
Day 0 before lysis	–	–	–	–	3
Day 1 postlysis	–	–	–	–	3
Day 3 postlysis	–	–	–	–	0
Day 7 postlysis	–	–	–	–	0
Day 12 postlysis		–	–	–	0
Vasopressin (U/min)					
Day 0 before lysis	0.04	0.04	0.04	0.04	–
Day 1 postlysis	0.04	0.04	0.04	0.04	–
Day 3 postlysis	0.04	0	0.04	0	–
Day 7 postlysis	0	0.04	0	0	–
Day 12 postlysis		0	0	0	–
Laboratory results					
hs‐Cardiac troponin (ng/L)					
Day 0 before lysis	0.54	0.35	1.63	0.99	3.3
D‐Dimmer (ng/ml)					
Day 0 before lysis	3788	5156	7172	1212	4003
Day 1 postlysis	1484	8291	6324	1630	6242
Day 3 postlysis	972	4738	2000	1090	5520
Day 7 postlysis	613	1331	1165	890	3335
Day 12 postlysis		864	682	1036	1100
Serum ferritin (ng/ml)					
Day 0 before lysis	619	1006	1265	761	1030
Day 1 postlysis	853	1186	843	1235	4227
Day 3 postlysis	576	791	820	1550	2246
Day 7 postlysis	382	476	360	1443	551
Day 12 postlysis		311	269	996	493
Lactate dehydrogenase (U/L)					
Day 0 before lysis	995	1167	795	615	415
Day 1 postlysis	676	1003	798	557	666
Day 3 postlysis	597	830	571	690	679
Day 7 postlysis	626	485	496	583	891
Day 12 postlysis		302	342	601	294
Procalcitonin (ng/ml)					
Day 0 before lysis	2.98	1.6	0.6	4.3	3.5
Day 1 postlysis	3.19	1.1	3.18	4.5	4.6
Day 3 postlysis	2.61	1.8	2.16	4.1	4.0
Day 7 postlysis	1.32	1.2	0.7	4.8	2.21
Day 12 postlysis		0.6	0.2	2.4	1.0

MAP, mean arterial pressure; P/F ratio, PaO_2_/FiO_2_ ratio; SOFA score, sequential organ failure assessment score.

### 3.2 Change in PaO_2_/FiO_2_ values

All five patients were receiving mechanical ventilation at the time of lysis therapy administration, and all patients were extubated after receiving the lysis therapy protocol within a time period of 2 weeks. Patient 5 was receiving ECMO at the time of lysis therapy initiation but did not require ECMO within 6 days postlysis. Prior to receiving the lysis therapy, the PaO_2_/FiO_2_ values of all patients were below 300 and ranged from 107 to 211. Upon administration of the lysis therapy, the PaO_2_/FiO_2_ values of all patients gradually increased over 350 within a time period that ranged from 7 to 12 days (Table 2, Figure 1B).

### 3.3 Change in SOFA score

The SOFA score ranged from +6 to +10 prior to receiving lysis therapy and decreased to a range of +1 to +5 at the twelfth day (the seventh day for Patient 1) following administration of lysis therapy (Table 2 and Figure 1C). The SOFA score is calculated using six systems: respiratory, coagulation, hepatic, cardiovascular, central nervous system, and kidney; and the worst value on each day was recorded.

### 3.4 Change in laboratory markers and body temperature

All five patients had high‐sensitivity cardiac troponin levels that ranged from 0.35 to 3.3 ng/L at the time of lysis therapy administration. The values of D‐dimmer were significantly high in all five patients prior to lysis therapy (a value less than 250 ng/ml is considered normal); these values markedly decreased postlysis within 7 to 12 days, except for Patient 4 who showed a mild variation. The serum levels of inflammatory markers such as serum ferritin, lactate dehydrogenase, and procalcitonin eventually decreased, but not normalized, within 7 to 12 days postlysis, except for Patient 4 who showed mild variations (Table 2). Body temperature ranged from 37.2 to 39.0°C prior to lysis therapy and declined to the normal ranges on the third‐day postlysis, except for Patient 2 which regained fever by day 2 postlysis which then eventually normalized by the eleventh‐day postlysis (Table 2 and Figure 1D).

### 3.5 Change in chest imaging

Chest x‐rays of these five patients demonstrated severe pneumonia prior to lysis therapy and showed gradual resolution of pulmonary lesions after administration of lysis therapy within a time period of 2 weeks (Supplementary figures).

### 3.6 Safety and adverse effects

The implemented low‐dose protocol of alteplase seems to be effective and well tolerated. All five patients did not suffer significant side effects or bleeding.

## 4 DISCUSSION

In this report, thrombolytic therapy was used to treat five hemodynamically unstable, mechanically ventilated COVID‐19 patients who showed echocardiographic signs of right ventricular strain. This therapeutic intervention showed a durable result in the form of restoring hemodynamic stability and increasing the ventilation capacity of the treated patients. The clinical conditions of these patients improved, as indicated by restoring circulatory rigor, improved PAO2/FIO2, decreased SOFA score, body temperature reduction, and chest imaging. All treated patients, who had been receiving mechanical ventilation and ECMO, were no longer in need of respiratory or circulatory support, and then transferred to the ward within days of receiving the lysis therapy.

The rationale for use of thrombolytic therapy in critical COVID‐19 patients is straightforward. Patients diagnosed with COVID‐19 infection are at increased risk for developing thrombotic vascular occlusions and the histopathological examination often reveals fibrin‐based obstructions in the lungs’ small vasculature of patients who succumb to severe forms of the disease [13, 16]. COVID‐19 is a newly emerged viral infection and studies that implemented lysis therapy in the treatment of the disease are scarce. Multiple case studies, which recruited hospitalized patients with COVID‐19 infection, reported an improvement of patients’ oxygenation after using thrombolytic therapy [17, 18]. A retrospective cohort study of 12 decompensated patients with severe COVID‐19 treated using alteplase concluded that the mortality rate decreased from 88% to 41.6%, which represents a significant improvement in patients requiring advanced respiratory support [19]. These studies have methodological limitations including unclear patient selection protocols, lack of reporting for patient baseline characteristics, inadequate duration of follow‐up, and partial reporting of outcomes. Our study improves the methodological limitations in the previous literature by giving clear indications and inclusion criteria for patients eligible to receive lysis therapy, reports patients’ baseline characteristics prior to lysis therapy administration, defines the dosing and therapeutic protocol, and delineates detailed clinical outcome postlysis therapy with adequate follow‐up duration.

All enrolled patients did not receive antiviral agents and the SARS‐CoV‐2 virus was still detectable in all 5 patients at the time of extubation. Therefore, the study results highlight the possibility that lysis therapy has contributed to the observed clinical improvement.

### 4.1 Recommendations

The main recommendations of this report are the following: (i) bedside echocardiography can be a beneficial tool in the urgent assessment of the right ventricle‐to‐pulmonary vascular coupling in mechanically ventilated COVID‐19 patients and (ii) a low‐dose protocol of lysis therapy may be beneficial in mechanically ventilated, hemodynamically unstable COVID‐19 patients who show echocardiographic criteria of right ventricular strain. The decision of implementing lysis therapy should weigh the current mortality rate of this patient subset in the absence of a specific antiviral treatment, besides the proposed benefits of clinical improvement against the possible bleeding complication of such intervention.

### 4.2 Limitations

This study has following limitations: (i) a case series included no controls and (ii) alteplase was administered immediately after fulfilling the patient inclusion criteria; whether a different timing of administration would have been associated with different outcomes cannot be determined.

## 5 CONCLUSION

In this case‐series of five mechanically ventilated, hemodynamically unstable COVID‐19 patients who show echocardiographic criteria of right ventricular strain, administration of low‐dose protocol of alteplase as a lysis therapy was followed by improvement of the patients’ clinical parameters. The limited sample size and study design preclude a definitive statement about the potential effectiveness of this treatment, and these observations require further evaluation in clinical trials.

## CONFLICT OF INTEREST

The authors declare that the article was prepared in the absence of any commercial or financial relationships that could be construed as a potential conflict of interest.

## FUNDING

The authors state that this article received no funding.

## AUTHOR CONTRIBUTIONS

AM, RT, and NJ performed the therapeutic intervention. EA developed the theory, designed the study, and wrote the manuscript with support from AM and RT. HF revised the manuscript and supervised the study. All authors contributed equally to this work, and all authors have read and approved the manuscript.

## Supporting information

Supporting Information

Supporting Information

Supporting Information

Supporting Information

Supporting Information
