# Supplementary material for: Using lysis therapy to treat five critically ill COVID‐19 patients who show echocardiographic criteria of right ventricular strain
Source: EJHaem. 2021 Oct 13;2(4):799–804. doi: 10.1002/jha2.307 (PMC8661526; doi:10.1002/jha2.307)
Supplement: Supplementary file 4 — Supporting Information [file JHA2-2-799-s005.pdf]

DETAILED METHODS:

Laboratory-confirmed COVID-19 patients who had any of the following were considered in a critical condition: (i) shock, identified by implementation of vasopressor therapy and elevated lactate levels (>2 mmol/L) despite adequate fluid resuscitation, (ii) respiratory failure requiring mechanical ventilation, or (iii) failure of other organs necessitating admission to the intensive care unit.

Patients:

Patients, who were diagnosed with Covid-19 infection using a real-time reverse-transcriptase (RT) PCR (DiaPlexQ™, SolGent Co. LTD), were eligible to receive lysis therapy if they fulfilled the following criteria: (i) had a rapidly progressive severe pneumonia (ii) were currently supported by mechanical ventilation, (iii) PaO_2/FiO_2 ratio <300 (wherein PaO_2 measured in mmHg and FiO_2 is the fraction of inspired oxygen expressed as a decimal), and (iv) the echocardiographic examination showed one or more criterion of right ventricular strain which comprises right ventricular (RV) dilatation > 1:1 of Left ventricle (LV) size, RV systolic dysfunction, mid-RV wall hypokinesis with apical sparing (McConnell's sign), moderate to severe tricuspid regurgitation, paradoxical septal wall motion deviation towards the LV, pulmonary artery dilatation, atrial dilatation, right heart thrombus or thrombus in transition, lack of respiratory variation of the inferior vena cava, and/or right ventricular outflow tract acceleration time (AT <60 ms) with a pulmonary arterial systolic pressure (PASP) of less than 60 mmHg (60/60 sign).

Real-Time PCR:

The nasopharyngeal swabs collected during hospitalization were sent to the laboratory in a viral transport case. Nucleic acid extraction from the samples was performed using the QIAamp™ viral RNA mini-kit (Qiagen Co.), and a quantitative real-time reverse-transcriptase PCR (RT-qPCR) was

performed using a commercial kit specific for 2019-nCoV detection (DiaPlexQ™, SolGent Co. LTD) approved by the United States Food and Drug Administration (FDA). Each RT-qPCR assay provided a cycle threshold (Ct) value, which is defined as the number of cycles required for the fluorescent signal to cross the threshold. Ct levels are inversely proportional to the amount of target nucleic acid in the sample. The specimens were considered positive if the Ct value was equal or less than 37 and negative if the results were undetermined[1]. Specimens with a Ct value higher than 37 were repeated and considered positive if the repeated results were the same as the initial results and ranged from 37 to 40. Whereas the specimen was considered negative if the repeated Ct was undetectable. All procedures involving the clinical specimens were performed in a biosafety level 3 laboratory.

Lysis Therapy:

A lower-dose protocol of recombinant tissue-type plasminogen activator[2] was implemented, wherein Alteplase (Activase®, Genentech) was intravenously infused over 15 minutes with a dose of 0.6 mg/kg (the maximum dose is 50 mg)[3]. Alteplase induces fibrinolysis by binding to fibrin in a blood clot and activates the clot-bound plasminogen via cleaving the Arg561-Val562 peptide bond to form plasmin[4]. Plasmin is a fibrinolytic enzyme that cleaves the cross-links between polymerized fibrin molecules, causing the blood clot to break down and dissolve[5]. Absolute contraindications of using lysis therapy are previous intracranial hemorrhage, recent ischemic stroke within 6 months, structural cerebral vascular lesion, intracranial neoplasm, aortic dissection, active bleeding, significant head injury, recent Intracranial or spinal surgery, or severe uncontrolled hypertension. Relative contraindications are active peptic ulcer, pregnancy, internal bleeding within 2 to 4 weeks, noncompressible vascular punctures, or advanced liver disease[6–8]. The most serious side-effect of lysis therapy is bleeding which can be life-threatening with an ominous prognosis[9]. Major bleeding

is defined as fatal bleeding or overt bleeding with a drop in haemoglobin level of at least 20 g/L or requiring transfusion of at least 2 units packed blood cells, or hemorrhage into a critical anatomical site (such as intracranial, intraspinal, intraocular, retroperitoneal, or pericardial)[10,11]. While minor bleeding is defined as any acute or subacute clinically overt bleeding that did not satisfy the criteria for major bleeding.

Clinical Information:

Clinical information for the five patients before and after lysis therapy was obtained from a review of the hospital computerized medical system and included the following: the demographic data, history of chronic diseases, days of admission from symptom onset, and the presenting symptoms; data about the implemented treatments, including mechanical ventilation, immunomodulators, and steroids; the clinical data, comprising body temperature, blood pressure, respiratory and heart rates, PaO_2/FiO_2 ratio, O_2 saturation, and Sequential Organ Failure Assessment (SOFA) score (range 0-24, with higher scores indicating more severe illness); laboratory data, including complete blood count, chemistry panels assessing liver and kidney function, and inflammatory markers; data from chest imaging studies; and information on complications, such as superimposed bacterial pneumonia, acute respiratory distress syndrome (ARDS), and multiple organ failure syndrome.

References:

1 Yang Y, Yang M, Yuan J, Wang F, Wang Z, Li J *et al.* Laboratory Diagnosis and Monitoring the Viral Shedding of SARS-CoV-2 Infection. *Innov* 2020; **1**: 100061.

2 Zhang Z, Zhai Z, Liang L, Liu F, Yang Y, Wang C. Lower dosage of recombinant tissue-type plasminogen activator (rt-PA) in the treatment of acute pulmonary embolism: a systematic review and meta-analysis. *Thromb Res* 2014; **133**: 357–363.

3 Konstantinides S V, Torbicki A, Agnelli G, Danchin N, Fitzmaurice D, Galiè N *et al.* 2014 ESC Guidelines on the diagnosis and management of acute pulmonary embolism: The Task Force for the Diagnosis and Management of Acute Pulmonary Embolism of the European Society of Cardiology (ESC) Endorsed by the European Respiratory Society (ERS). *Eur Heart J* 2014; **35**: 3033–3080.

4 Mican J, Toul M, Bednar D, Damborsky J. Structural biology and protein engineering of thrombolytics. *Comput Struct Biotechnol J* 2019; **17**: 917–938.

5 Gurman P, Miranda OR, Nathan A, Washington C, Rosen Y, Elman NM. Recombinant tissue plasminogen activators (rtPA): a review. *Clin Pharmacol Ther* 2015; **97**: 274–285.

6 O'Gara PT, Kushner FG, Ascheim DD, Casey DE, Chung MK, De Lemos JA *et al.* 2013 ACCF/AHA guideline for the management of ST-elevation myocardial infarction: a report of the American College of Cardiology Foundation/American Heart Association Task Force on Practice Guidelines. *J Am Coll Cardiol* 2013; **61**: e78–e140.

7 Ibanez B, James S, Agewall S, Antunes MJ, Bucciarelli-Ducci C, Bueno H *et al.* 2017 ESC Guidelines for the management of acute myocardial infarction in patients presenting with ST-

segment elevation: The Task Force for the management of acute myocardial infarction in patients presenting with ST-segment elevation of the European Socie. *Eur Heart J* 2017; **39**: 119.

8 Fugate JE, Rabinstein AA. Absolute and relative contraindications to IV rt-PA for acute ischemic stroke. *The neurohospitalist* 2015; **5**: 110–121.

9 Tisdale JE, Colucci RD, Ujhelyi MR, Kluger J, Fieldman A, Chow MSS. Evaluation and comparison of the adverse effects of streptokinase and alteplase. *Pharmacother J Hum Pharmacol Drug Ther* 1992; **12**: 440–444.

10 Schulman S, Kearon C, Haemostasis S on C of A of the S and SC of the IS on T and. Definition of major bleeding in clinical investigations of antihemostatic medicinal products in non-surgical patients. *J Thromb Haemost* 2005; **3**: 692–694.

11 Roskell NS, Samuel M, Noack H, Monz BU. Major bleeding in patients with atrial fibrillation receiving vitamin K antagonists: a systematic review of randomized and observational studies. *Europace* 2013; **15**: 787–797.
