# Supplementary material for: Using lysis therapy to treat five critically ill COVID‐19 patients who show echocardiographic criteria of right ventricular strain
Source: EJHaem. 2021 Oct 13;2(4):799–804. doi: 10.1002/jha2.307 (PMC8661526; doi:10.1002/jha2.307)
Supplement: Supplementary file 5 — Supporting Information [file JHA2-2-799-s001.pdf]

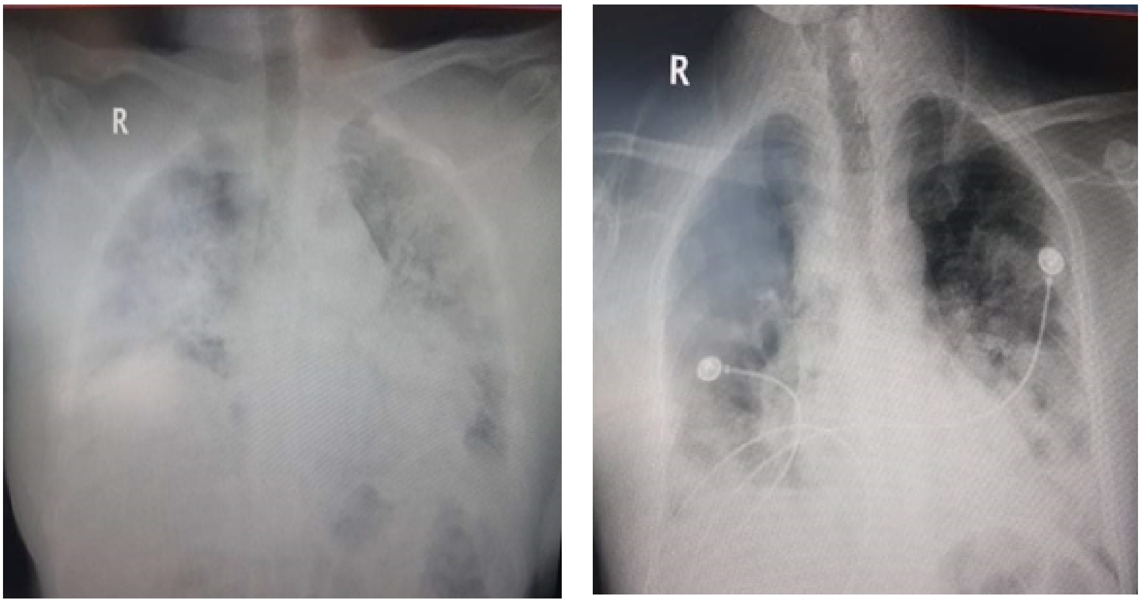

Chest X-Ray of Patient 1: At time of ICU addmission (on the left) and at time of extubation (at the right).

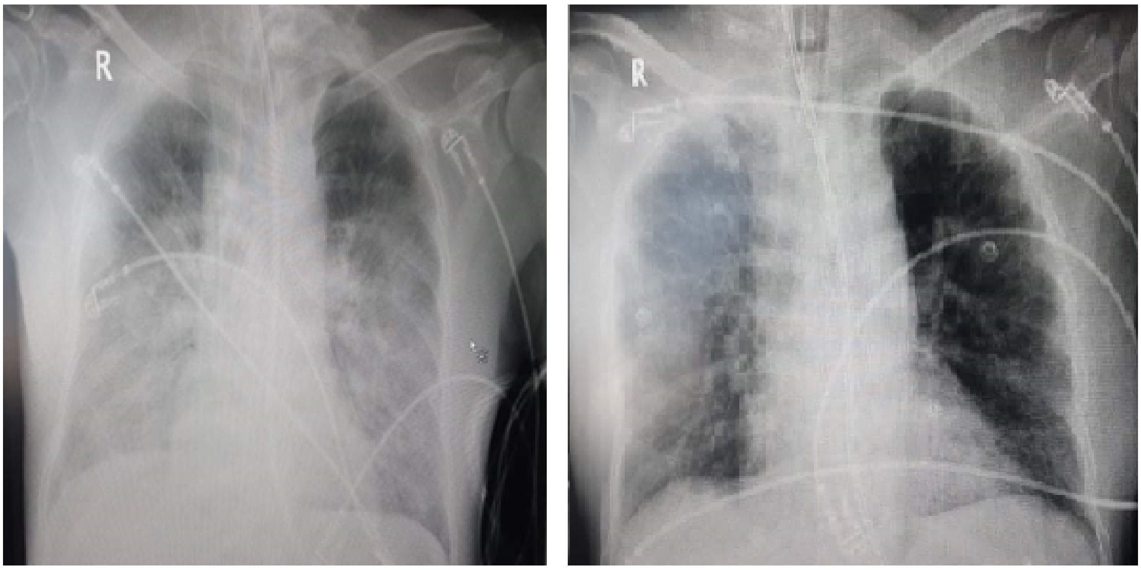

Chest X-Ray of Patient 2: At time of ICU addmission (on the Left) and at time of extubation (on the right).

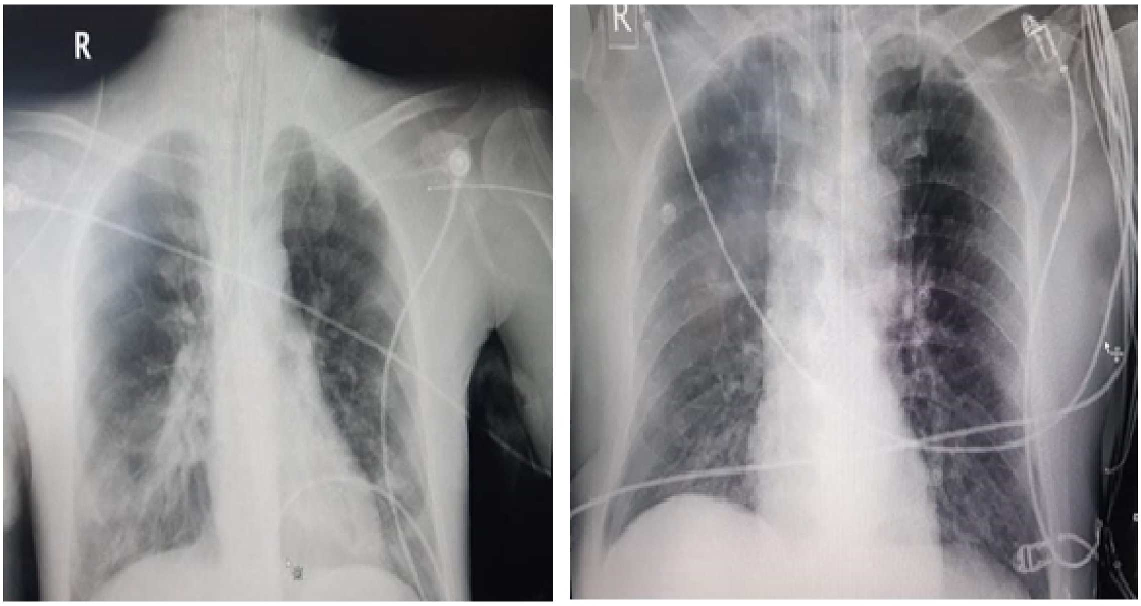

Chest X-Ray of Patient 3: at time of ICU addmission (on the left) and at time of extubation (on the right).

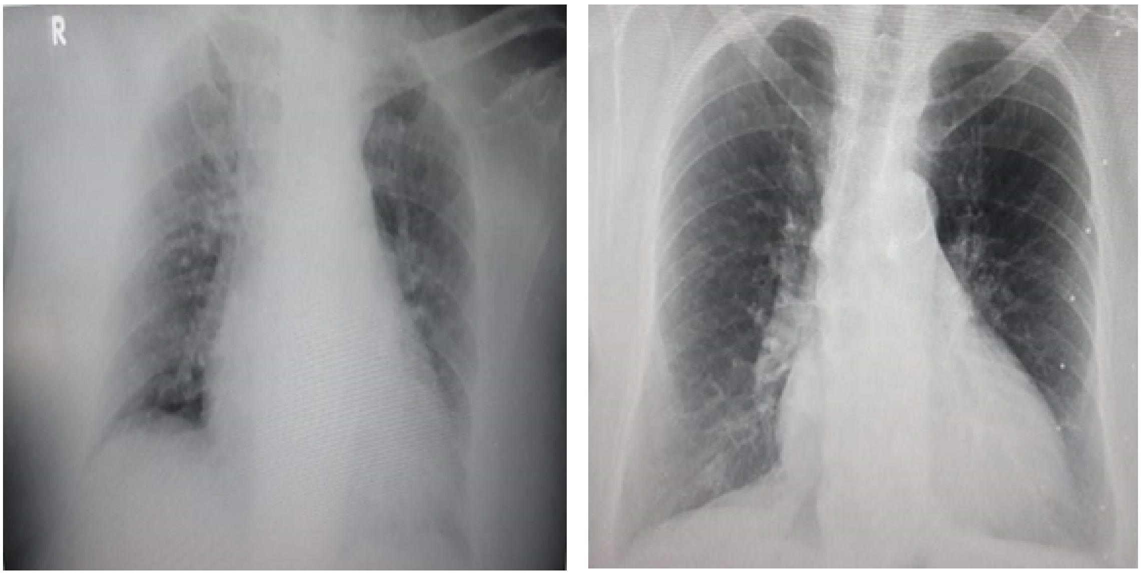

Chest X-Ray of Patient 4: At time of addmission (on the left) and at time of Discharge (on the right).

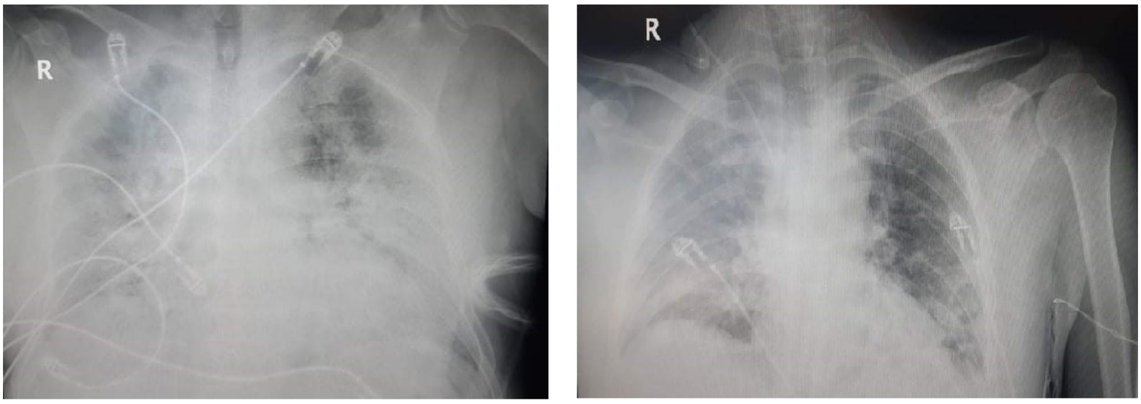

Chest X-Ray of Patient 5: At time of ICU addmission (on the left) and at time of extubation (at the right).
